# Lagrangian Tropical Cyclone Precipitation Estimates and Moisture Sources (LagTCPMoS) Dataset

**DOI:** 10.1038/s41597-025-05490-y

**Published:** 2025-07-08

**Authors:** Albenis Pérez-Alarcón, Ricardo M. Trigo, Raquel Nieto, Luis Gimeno

**Affiliations:** 1 Centro de Investigación Mariña, Universidade de Vigo, Environmental Physics Laboratory (EPhysLab), Campus As Lagoas s/n, 32004 Ourense, Spain; 2 Instituto Dom Luiz (IDL), Faculdade de Ciências, Universidade de Lisboa, 1749-016 Lisboa, Portugal; 3 Departamento de Meteorologia, Universidade Federal do Rio de Janeiro, Rio de Janeiro, 21941-919 Brazil; 4 Galicia Supercomputing Center (CESGA), Santiago de Compostela, Spain

**Keywords:** Atmospheric dynamics, Hydrology

## Abstract

This work presents a Lagrangian tropical cyclone (TC) precipitation estimates and moisture sources (LagTCPMoS) dataset in the North Atlantic basin from 1980 to 2023. TC tracks were obtained from the U.S. National Hurricane Center HURDAT2 dataset, and the Lagrangian precipitation estimates and moisture sources were computed by applying a Lagrangian moisture tracking approach to the global outputs of the FLEXible PARTicle (FLEXPART v10.4) dispersion model. The LagTCPMoS dataset has two main products for each 6-hourly track point along the TC tracks: the precipitation estimates over a fixed and symmetrical radius of 500 km and the spatial distribution of the origin of moisture for that precipitation at 0.5° × 0.5° grid spacing. We presented an overall assessment and the validation of the Hurricane Harvey (2017) case against the high-resolution Multi-Source Weighted-Ensemble Precipitation (MSWEP) dataset to illustrate that both the Lagrangian precipitation estimates and moisture uptake are well captured.

## Background & Summary

Heavy precipitation associated with tropical cyclones (TCs) can cause significant damage, including human and economic losses, in both tropical and subtropical regions worldwide^1–5^. However, TC-related precipitation (TCP) is a double-edged sword, as it often represents not only a serious hazard but also an important water supply mechanism^6,7^. On this basis, TCs can contribute a substantial amount of precipitation, which may represent a significant fraction of the annual precipitation in coastal regions. For example, TCs account for ~10–30% of precipitation over East Asia^8^, ~11% in Cuba^9^, ~8–12% along the southeastern coast of the United States^10^ and ~35–50% of the annual precipitation in the northern Philippines, Baja California (Mexico), northwestern Australia and southeastern China^11^. A reliable TCP estimate based on observational data is key to understanding the impact of TCs on local and global water budgets. In this context, Morin *et al*.^12^ recently developed a global multi-source TCP dataset based on the high-resolution Multi-Source Weighted-Ensemble Precipitation (MSWEP) database^13^.

Similarly, understanding the contribution of moisture source and transport pathways can contribute to understanding the variability and origin of precipitation, including TCP^6,14,15^. This task is addressed by applying numerous moisture-tracking tools^14^, e.g., analytical^16^, Eulerian^17^ and Lagrangian^18–20^ models. In particular, Lagrangian moisture tracking approaches^21–23^ based on the water budget equation^21,22^ have recently been widely used to investigate the origin of moisture for TCP^7,24–29^ because they provide spatial and temporal insights into the moisture pathways. However, the resulting datasets for the moisture source patterns and Lagrangian precipitation estimates are either not publicly available or are not regularly updated.

Therefore, this work presents a Lagrangian TCP estimates and moisture sources (LagTCPMoS) dataset derived from the global FLEXible PARTicle (FLEXPART v10.4) dispersion model^20^ simulations using 30 million air parcels^30^ and the Lagrangian Atmospheric moiTture and heaT trackINg (LATTIN) tool^31^. The current version of LagTCPMoS includes the 6-hourly Lagrangian TCP estimates over a fixed and symmetrical radius of 500 km^11,12,32,33^ and the moisture uptake for all TCs formed in the North Atlantic basin from 1980 to 2023. The bias in these quantities has also been corrected using a simple bias correction approach^34^ based on the MSWEP dataset. LagTCPMoS is provided in a user-friendly format using NetCDF files. It is also worth noting that LagTCPMoS is closely related to the widely used Atlantic hurricane database (HURDAT2)^35^ supported by the U.S. National Hurricane Center, as it takes the TC position from the HURDAT2 dataset.

Fully validating the resulting patterns of precipitation and moisture sources contributions is challenging for the Moisture Tracking Community^36^ due to the scarcity of observations. On this basis, LagTCPMoS offers new research opportunities. For example, it can serve to benchmark other moisture tracking tools or machine learning approaches focused on investigating the origin and quantifying TC precipitation, which is a further step in reducing uncertainties in the moisture sources analysis. In addition, we aim to expand this dataset to cover the other ocean basins in future updates and maintain its currency.

## Methods

### Input data

TC tracks were retrieved from the Atlantic hurricane database (HURDAT2)^35^. HURDAT2 entries include TC position in space (latitude and longitude) and time, maximum wind speed, and minimum sea level pressure.

The global simulations of FLEXPART (v10.4) model^20^ were performed using 30 million air parcels of equal mass^30^. The model was fed by the 3-hourly meteorological data from the European Centre for Medium-Range Weather Forecasts ERA5 reanalysis^37^ at 0.5° × 0.5° horizontal resolution and 137 vertical levels. FLEXPART uses the Emanuel and Živković-Rothman^38^ convection scheme to account for subgrid-scale convective transport, the Hanna^39^ boundary layer turbulence scheme and the Cassiani *et al.*^40^ skewed turbulence option

The Multi-Source Weighted-Ensemble Precipitation (MSWEP) database^13^ is a global 3-hourly precipitation product with 0.1° × 0.1°grid spacing available since 1979. It merges data from several sources such as gauge stations, satellites and reanalysis intending to achieve the best quality by timescale and location. MSWEP was used as a reference for bias-correcting the Lagrangian TCP estimates and the moisture uptake patterns.

### Lagrangian precipitation estimate

**Fig. 1:**
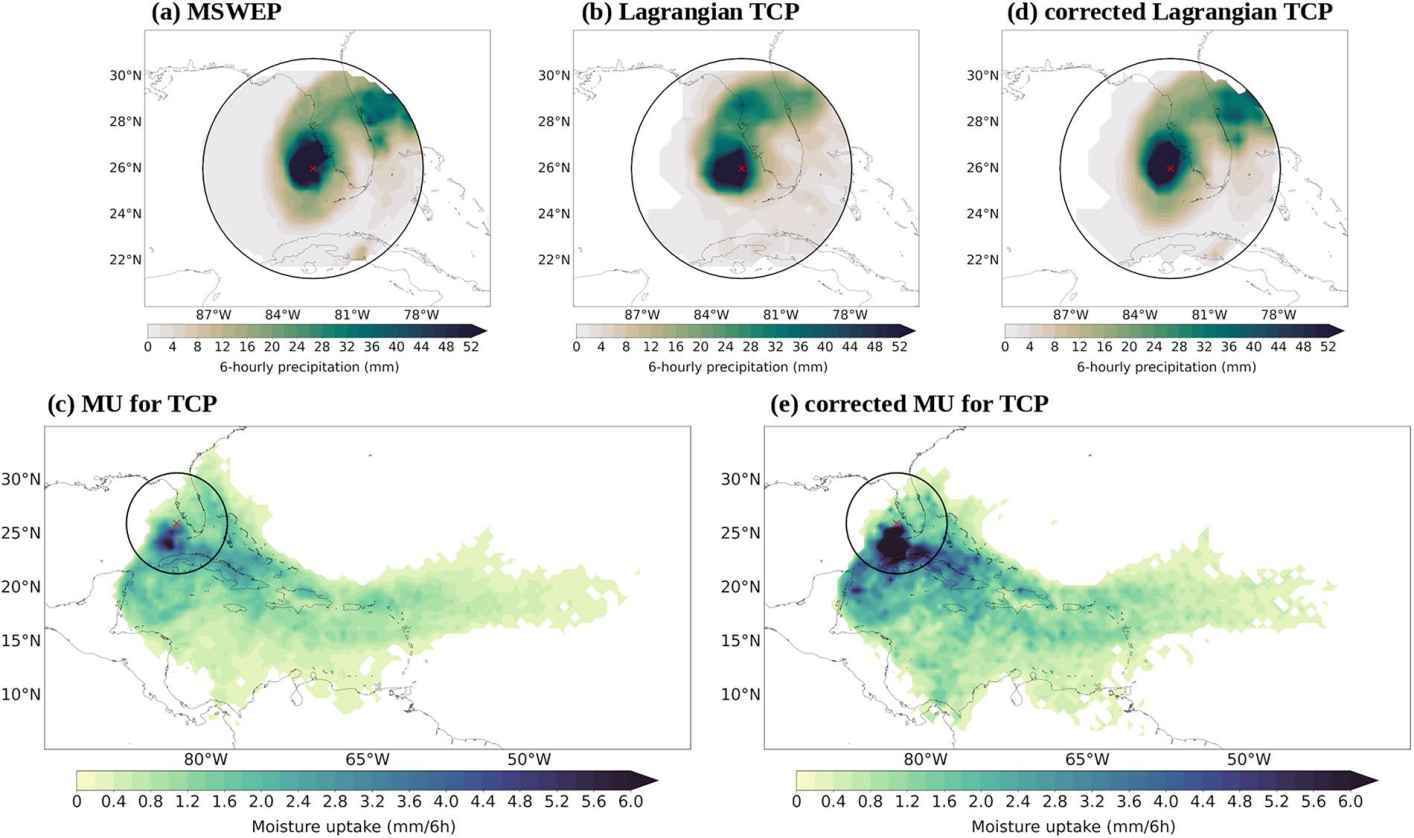
Example of the Lagrangian tropical cyclone 6-hourly precipitation (TCP) estimate and moisture uptake (MU) for the case of Hurricane Ian on 28 September 2022 at 1200 UTC. TCP from (**a**) Multi-Source Weighted-Ensemble Precipitation (MSWEP) database, (**b**) Lagrangian TCP and (**d**) bias-corrected Lagrangian TCP. Panels (**c**) and (**e**) illustrate the moisture uptake and bias-corrected moisture uptake, respectively. The black circle and red cross denote the area enclosed by the 500 km radii and the TC centre, respectively.

According to the Lagrangian water budget equation^21,22^, an air parcel can gain or lose moisture through evaporation or precipitation, respectively. On this basis, by assuming that moisture losses immediately fall as precipitation^23^, the precipitation at the surface over the target region (defined as the area enclosed by a 500 km radial distance from the TC centre) on a 0.5° × 0.5° regular grid can be estimated as follows:

1 $${TCP}=\frac{m}{A}\sum {\triangle q}_{k}^{0}$$

where *m* is the air parcel mass, *A* is the area of the target region, and ∆*q*^0^ is the specific humidity decrease (∆q^0^ < 0 g/kg) in the last 6 hours before the air parcels reached the TC. Only those air parcels with relative humidity larger than 65% are considered for accounting for TCP^34^. As an example, Fig. 1a,b display the 6-hourly TCP for the case of Hurricane Ian on 28 September 2022 at 1200 UTC from the MSWEP and Lagrangian estimates, respectively.

It is worth noting that TCP distribution is determined by many factors, such as wind shear, sea surface temperatures, moisture distribution, and TC-specific intensity, location, and translation speed^41–43^. Therefore, a fixed and symmetrical radius of 500 km may be too large or too small in some cases. However, we chose 500 km from the TC centre because it has been widely used in previous studies as a proxy for the radius of TCP^11,12,32,33,44,45^.

### Lagrangian moisture sources analysis

From the Lagrangian point of view, the moisture changes in an air parcel reflect the effects of both evaporation and precipitation^21,22^ during its movement throughout the atmosphere. Therefore, to identify the regions that are contributing moisture for TCP, we backtracked air parcel trajectories that precipitated within the TC (see the Lagrangian precipitation estimate section) for each TC location for up to 10 days. Air parcel pathways were extracted from the global FLEXPART model simulations^30^. Meanwhile, a trajectory length of 10 days is chosen because it is widely accepted as the mean water vapour residence time in the atmosphere^46–48^, and it is also appropriated for investigating moisture sources for TCs based on their mean water vapour residence time of ~2-3 days^49^.

Following Pérez-Alarcón *et al*.^34^, if along the air parcel trajectory during a 6-hourly time interval, the specific humidity increases (∆q > 0 g/kg) and the relative humidity change is lower than 20%, a moisture uptake event is defined. On the contrary, if the specific humidity decreases (∆q < 0 g/kg) and the relative humidity is higher than 65%, a precipitation event in route occurs. Other changes in the moisture content of the air parcel are considered “non-physical processes” (e.g., numerical diffusion and numerical errors in the discretization of the differential equations in the Lagrangian models) and thereby discarded^34^. Precipitation in route is related to all previous moisture uptake events. For this reason, the amount of moisture losses is then discounted in proportion following Sodemann *et al*.^23^. Finally, the spatial pattern of moisture uptake (MU) on a 0.5° × 0.5° regular grid is computed by aggregating all resulting moisture changes in the atmospheric column of area A (see an example on Fig. 1c).

2 $${MU}=\frac{m}{A}\sum {\triangle q}_{k}$$

Further details on the moisture source analysis can be found in the original paper of Sodemann *et al*.^23^ and Pérez-Alarcón *et al*.^34^. This moisture tracking approach has been coded into the Lagrangian Atmospheric moisTure and heaT trackINg (LATTIN) tool^31^.

### Bias correction approach

To reduce uncertainties in the Lagrangian TCP estimates and moisture uptake pattern, we applied the simple bias correction approach proposed by Pérez-Alarcón *et al*.^34^, using the MSWEP dataset as a reference of the observational TCP. This methodology firstly computes the bias in the precipitation estimate (*∆TCP*_*i*_) in the target region over each atmospheric column of area *A*_*i*_ as follows:

3 $$\Delta {{TCP}}_{i}={{LagTCP}}_{i}-{R}_{i}$$

where R_i_ represents the TCP from the MSWEP dataset. Next, the moisture decreases ($c\Delta {q}_{i,k}^{0}$) in the target region is bias-corrected as follows:

4 $${c\Delta q}_{i,k}^{0}=|{\Delta q}_{i,k}^{0}|-{\Delta TCP}_{i}\cdot \frac{|{\Delta q}_{i,k}^{0}|}{\sum |{\Delta q}_{i,k}^{0}|}\cdot \frac{{A}_{i}}{m}$$

Then, the bias-corrected Lagrangian TCP (*cLagTCP*; see Fig. 1d) can be estimated by applying Eq. (1) to $c\Delta {q}_{i,k\,}^{0}$. Second, to correct the moisture uptake along the air parcel trajectories, the biased trajectories are removed and the moisture contributions to the final precipitation are adjusted following Eq. (5).

5 $$c\Delta {q}_{{t}_{j}}=\Delta {q}_{{t}_{j}}-\frac{\Delta {q}_{{t}_{j}}}{\sum {\Delta qt}_{i}}\cdot (\sum \Delta {q}_{{t}_{i}}-c\Delta {q}^{0})\,{\rm{f}}{\rm{o}}{\rm{r}}\,{\rm{a}}{\rm{l}}{\rm{l}}\,\Delta {q}_{{t}_{i}} > \Delta {q}_{mu}$$

where t_j_ represents the parcel location from the end to the starting point. Third, the bias-corrected MU (cMU’) pattern is computed by combining Eqs. (2, 5). Finally, given the differences in the grid cell area (A_i_), the total bias-corrected moisture uptake could be different from the bias-corrected Lagrangian TCP. Therefore, to guarantee that the total cMU matches *cLagTCP*, we applied a final adjustment (Eq. 6)^34^. This bias-correction approach has been implemented in the LATTIN tool. Figure 1e shows an example of the cMU pattern for the case of Hurricane Ian on 28 September 2022 at 1200 UTC.

6 $${{cMU}}_{i}={{cMU}}_{i}^{{\prime} }-\frac{{{cMU}}_{i}^{{\prime} }}{\sum {cMU}{\prime} }(\sum {cMU}{\prime} -\sum {cLagTCP})$$

## Data Records

The LagTCPMoS^50^ is freely available for download at 10.5281/zenodo.14889141. This dataset is stored in a NetCDF file for each TC, containing, among others, the 6-hourly Lagrangian TCP estimates and moisture uptake. Each NetCDF file has the necessary metadata, and can be read using the common analysis software and programming languages. Table 1 shows the full list of variables in the LagTCPMoS dataset.

**Table 1:** Full list of variables in the LagTCPMoS dataset.

Variable name	Dimensions	Description
LagTCP	time, lat, lon	Lagrangian TCP estimate (mm/6 h)
cLagTCP	time, lat, lon	Bias-corrected Lagrangian TCP estimate (mm/6 h)
MoistureUptake	time, lat, lon	Moisture uptake (mm/6 h)
cMoistureUptake	time, lat, lon	Bias-corrected moisture uptake (mm/6 h)
TCmask	time, lat, lon	Mask of TC area over a fixed and symmetrical radius of 500 km
latc	time	Latitudes of TC centre
lonc	time	Longitudes of TC centre

Latitudes and longitudes of the TC centre are extracted from the HURDAT2 dataset. The first dimension (“time”) represents the temporal information of each 6-hourly TC position. The second dimension (“lat”) represents the latitudes, going northward from −90° to 90° at 0.5° intervals. Meanwhile, the third dimension (“lon”) informs about the longitudes, going eastward from −180° to 179.5° at 0.5° interval.

## Technical Validation

### The case of Hurricane Harvey

**Fig. 2:**
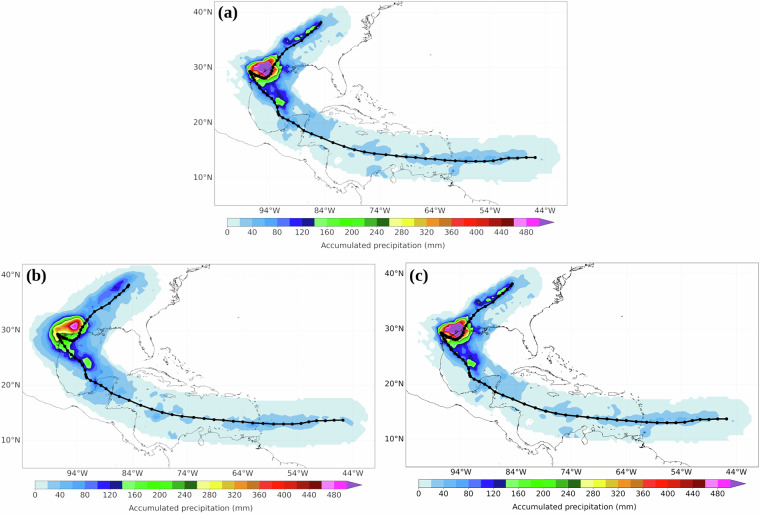
Hurricane Harvey accumulated precipitation over the 6-hourly track points from (**a**) MSWEP, (**b**) Lagrangian TCP and (**c**) bias-corrected Lagrangian TCP. The dark line denotes the Harvey track from 16 August to 2 September 2017.

To illustrate the reliability of LagTCPMoS, Fig. 2 shows the 6-hourly accumulated TCP along the Hurricane Harvey track. Hurricane Harvey formed in the North Atlantic basin from 16 August to 2 September 2017, reaching the major hurricane (category 3+) strength on the Saffir-Simpson wind scale^51^ and causing record levels of precipitation in the Houston (Texas, U.S) metropolitan area^52^. The accumulated Lagrangian TCP footprint from MSWEP reveals a good agreement with the estimate from Lagrangian TCP (Fig. 2a,b), but much better with the bias-corrected Lagrangian TCP (Fig. 2c). This different level of similitude is well encapsulated by computing the Spearman correlation coefficient of 0.84 and 0.99 (p < 0.05) and the Kling–Gupta efficiency (KGE)^34,53,54^ of 0.83 and 0.99 for the estimated and bias-corrected Lagrangian TCP, respectively, against the MSWEP. It is worth noting that KGE values closest to one indicate a good simulation. Likewise, there is good agreement with the spatial distribution and total Harvey precipitation reported in the literature^12,25,52,55^. Overall, this result demonstrates the ability of LagTCMoS, and consequently of the Lagrangian approach, to estimate TCP.

**Fig. 3:**
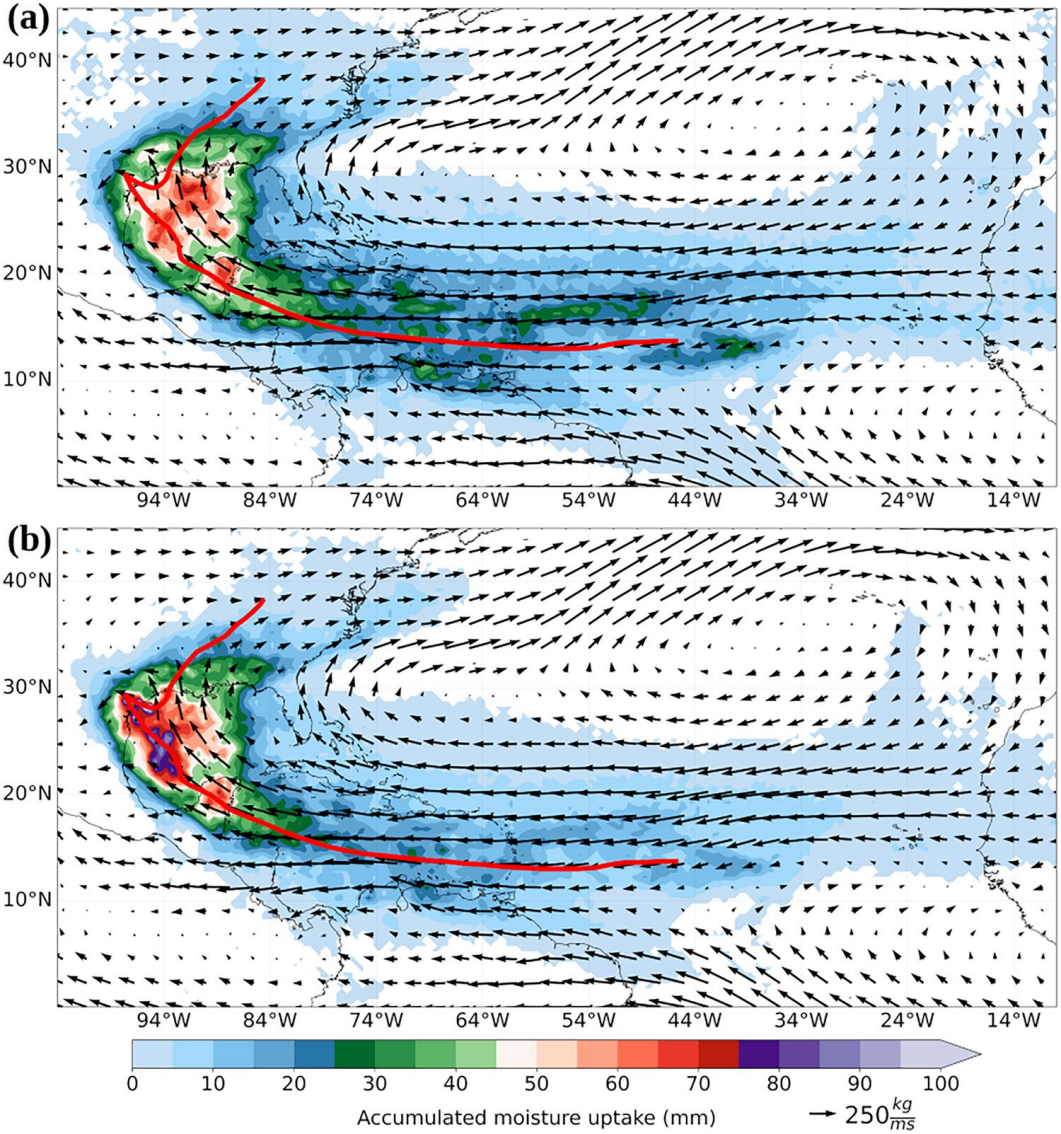
Hurricane Harvey accumulated moisture uptake during its lifecyle. (**a**) Moisture uptake and (**b**) bias-corrected moisture uptake. The solid red line denotes the Harvey track from 16 August to 2 September 2017. Arrows show the mean vertically integrated moisture flux (VIMF) (kg/ms) from the ERA5 reanalysis.

Figure 3 shows the 6-hourly accumulated moisture uptake for the precipitation produced by Hurricane Harvey during its lifecycle. The uncorrected (Fig. 3a) and bias-corrected (Fig. 3b) patterns reveal the Gulf of Mexico, followed by the Caribbean Sea, as the main moisture sources for Harvey precipitation. It is important to note the moisture contribution from the southeastern U.S. and more remote sources, such as the eastern North Atlantic Ocean, which is in line with previous studies^25,28^. Likewise, the highest moisture uptake occurs within 3–5° from the Harvey track, resembling the characteristics of moisture sources for TC precipitation in the North Atlantic basin^25,28^. Overall, the moisture transport is controlled by the circulation of the North Atlantic High-Pressure system and the easterly winds, as revealed by the mean vertically integrated moisture flux (VIMF) from the ERA5 reanalysis. Some differences in the moisture uptake between the uncorrected and bias-corrected patterns are observed, including the pattern intensity in the Gulf of Mexico and its eastward extension. TC Harvey made landfall twice (red line in Fig. 3) and impinged a tremendous amount of precipitation in the first landfall, thus providing an unusually high level of land moisture source for the following days, also owing to the fact that the TC progressed slowly close to the Texan shore. In this context, it is important to stress that the uncorrected MU pattern (Fig. 3a) shows a much weaker role of this moisture source over land than the bias-corrected MU version (Fig. 3b). These differences demonstrate the importance of the bias-correction approach in reducing uncertainties in the analysis of moisture sources for precipitation^56^, such as TCs^34^.

### Overall assessment

To further verify the quality of LagTCPMoS, we calculated several skill scores from a contingency table (Table 2): the probability of detection (POD = H/(H + M)), the probability of false detection (PFD = FA/(FA + CN)), the critical success index (CSI = H/(H + FA + M)), and Peirce’s skill score (PSS = POD-PFD). Additionally, we estimated the KGE index and the Spearman correlation coefficient between the observed precipitation from MSWEP and the Lagrangian precipitation estimates. A minimum 6-hour precipitation threshold of 1 mm was selected to distinguish between precipitation and no-precipitation events. This threshold value has been consistently applied in previous studies to assess the reliability of the Lagrangian approach in estimating precipitation^34,56^.

**Table 2:** Contingency table for assessing the accuracy of Lagrangian precipitation estimates compared to observed precipitation from the MSWEP data.

		Observed Precipitation	
		Yes	No
**Lagrangian precipitation estimates**	**Yes**	H: Hits	FA: False Alarms
	**No**	M: Misses	CN: Correct Negative

Hits: A precipitation event was successfully identified by the Lagrangian approach. Misses: Precipitation was present in the observations but overlooked by the Lagrangian estimation. False Alarms: The Lagrangian diagnosis detected precipitation where none was observed. Correct Negative: Accurate identification of no precipitation events.

**Fig. 4:**
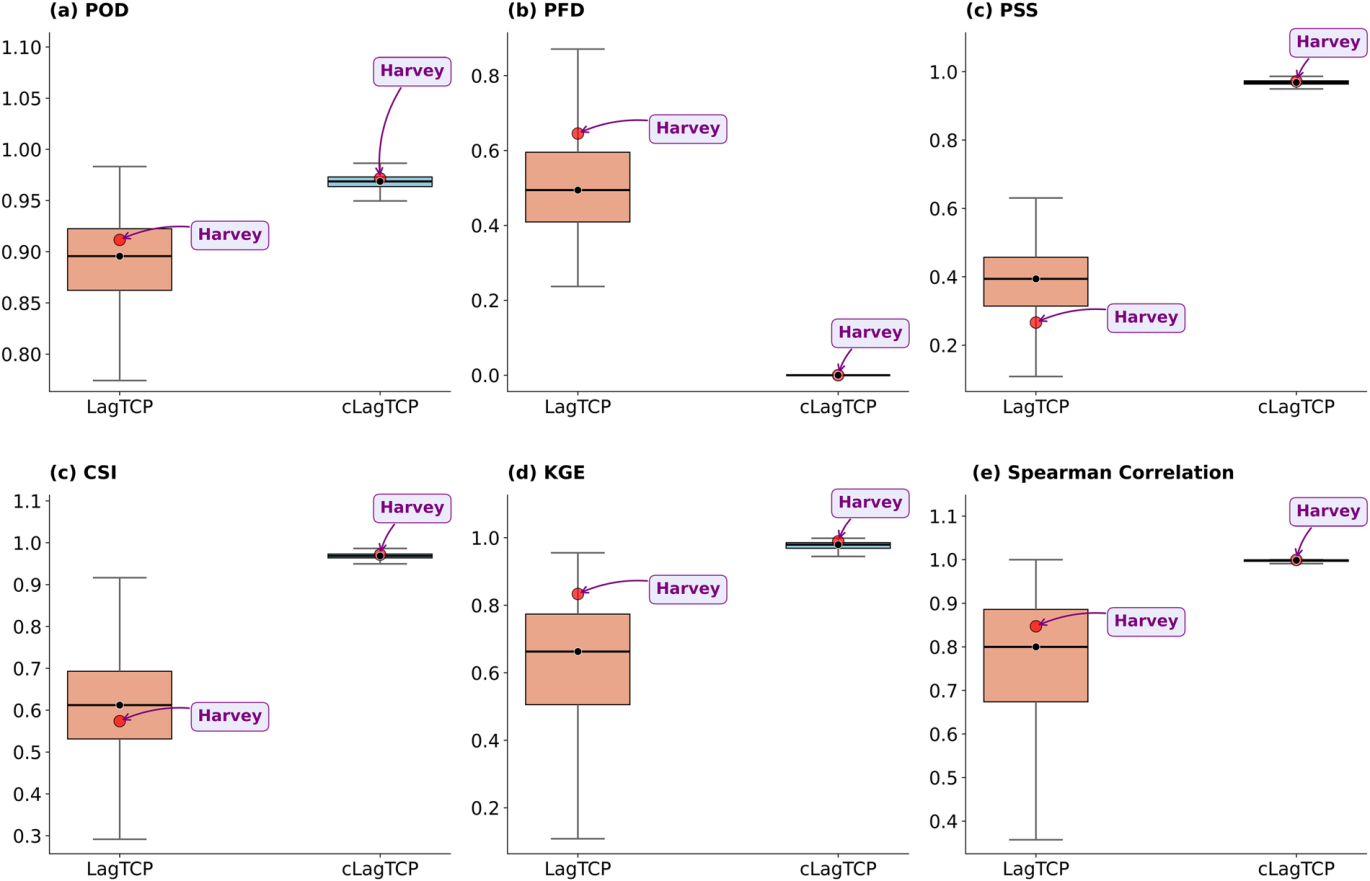
Performance evaluation of the Lagrangian (LagTCP) and bias-corrected Lagrangian (cLagTCP) precipitation estimates. Boxplots depict the distribution of (**a**) Probability of Detection (POD), (**b**) Probability of False Detection (PFD), (**c**) Peirce’s Skill Score (PSS), (**d**) Critical Success Index (CSI), (**e**) Kling-Gupta Efficiency (KGE), and (**f**) Spearman correlation coefficient. These metrics have been computed for each tropical cyclone in the dataset. Red marker highlights the statistics for the case of Hurricane Harvey.

Figure 4 presents the performance of Lagrangian precipitation estimates for the LagTCP and cLagTCP products compared to the MSWEP dataset. Across all evaluated metrics, the cLagTCP approach consistently demonstrates superior performance and reduced variability compared to LagTCP. For instance, cLagTCP exhibits markedly higher median values for the Probability of Detection (POD, ~0.97 vs. ~0.9; Fig. 4a), Peirce’s Skill Score (PSS, ~0.98 vs. ~0.4; Fig. 4c), Critical Success Index (CSI, ~0.97 vs. ~0.6; Fig. 4d), Kling-Gupta Efficiency (KGE, ~0.98 vs. ~0.65; Fig. 4e), and Spearman Correlation (~0.99 vs. ~0.8; Fig. 4f), indicating improved agreement with observations and higher overall skill due to the bias correction. This improvement is further corroborated by the significantly lower median Probability of False Detection (PFD, ~0.05 vs. ~0.5; Fig. 4b) for cLagTCP, representing a substantial reduction in false alarms. Figure 4 also provides these metrics for the specific case of Hurricane Harvey, supporting the results shown in Fig. 2. These performance levels, particularly for cLagTCP, are indicative of highly skilled precipitation estimation, consistent with previous studies assessing precipitation detection and estimation algorithms^34,57–59^.

**Fig. 5:**
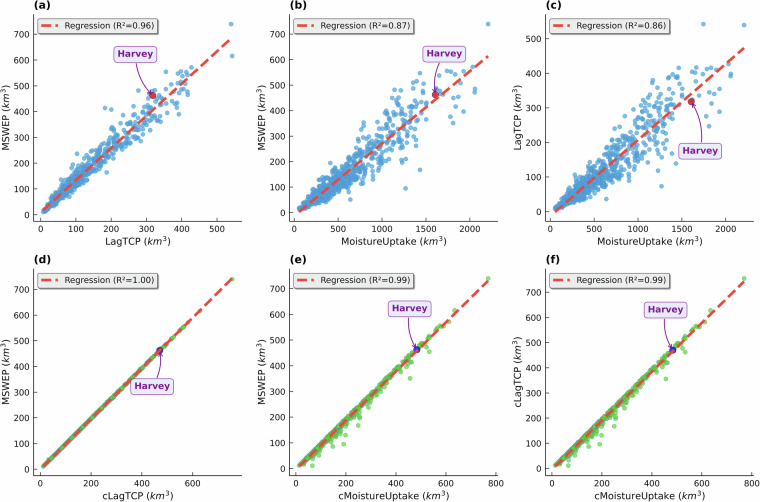
Scatter plots of the accumulated tropical cyclone-related precipitation from the MSWEP dataset and the Lagrangian estimates (LagTCP, cLagTCP) and their corresponding moisture uptake values (MoistureUptake, cMoistureUptake). Panels (**a**–**c**) show results for the raw Lagrangian approach, and panels (**d**–**f**) display relationships for the bias-corrected fields. The dashed red line denotes the regression line. Red (panels a–c) and blue (panels d–f) markers show the relationship for the case of Hurricane Harvey.

Figure 5 shows the relationships between accumulated TC-related precipitation from MSWEP, Lagrangian precipitation estimates (LagTCP), and the corresponding moisture uptake. Figure 5a reveals a strong agreement between MSWEP and LagTCP (R² = 0.96). However, when examining the relationships involving moisture uptake (Fig. 5b,c), a more dispersed pattern is evident in the scatter plots, although the determination coefficients remain above 0.85. This behaviour suggests that the moisture uptake tends to overestimate the precipitation, which can be attributed to inherent uncertainties of the Lagrangian diagnostic of moisture sources^15,20,60^. Notably, applying the bias correction approach leads to a noticeable improvement in these relationships. It is evident from the near-perfect linear fits and R² values approaching 1.0 across all bias-corrected fields (Fig. 5d–f), indicating high agreement. This result confirms the critical role of the bias-correction approach in reducing uncertainties in the Lagrangian moisture source identification^34,56^.

It is important to note that validating the accuracy of the spatial distribution of the moisture sources is a challenge for the Moisture Tracking Community^36^, primarily due to the scarcity of observations for benchmarking. Nonetheless, the overall assessment of the LagTCPMoS dataset indicates that the Lagrangian precipitation estimates and associated moisture sources reliably capture the precipitation of TCs in the North Atlantic basin.

### Limitations

Among the complex thermodynamic and dynamic processes involved in TCs and the known limitations of ERA5 reanalysis in resolving TCs and their surrounding environment due to its inability to capture some TC processes^61,62^, this dataset’s inherent uncertainties primarily stem from its reliance on Lagrangian moisture tracking analysis. Specifically, despite their overall robustness, Lagrangian tracking methodologies often struggle to accurately represent critical physical processes that influence atmospheric humidity, such as convection, turbulence, the evaporation of precipitating hydrometeors, subgrid-scale turbulent fluxes, or moist air subsidence. These processes are not directly captured or fully reflected by changes in humidity derived solely from bulk evaporation and precipitation. Other sources of uncertainty include factors like numerical diffusion, errors in trajectory calculations, the density of released air parcels, the chosen convection scheme, and inconsistencies between analysis time steps in Lagrangian models^15,20,60,63,64^. The overall impact of non-physical moisture losses in the air parcels before reaching the target region can be important when computing the moisture contributions from remote sources, because the errors are accumulated over time. Furthermore, the accuracy of bias-correction techniques is intrinsically linked to the reliability of the observed precipitation data used as reference^34^. Finally, it is also important to note that the precipitation patterns associated with TCs are substantially influenced by their translation speed and the direction of their trajectories^41–43,65^. These complexities underscore the need for continuous improvement in modelling approaches to enhance our understanding of the moisture sources for TC precipitation.

## Data Availability

The Lagrangian Atmospheric moisTure and heaT trackINg (LATTIN) tool^31^ used to computed the Lagrangian precipitation estimates and moisture uptake is accessible through its GitHub repository at https://github.com/apalarcon/LATTIN.
